# Extended-spectrum beta-lactamase producing *Enterobacteriaceae* (ESBL-E) isolated from bean sprouts in the Netherlands

**DOI:** 10.1371/journal.pone.0203338

**Published:** 2018-08-30

**Authors:** Pepijn Huizinga, Eefje Schrauwen, Silvia García-Cobos, Ina Willemsen, Carlo Verhulst, Alexander W. Friedrich, Paul H. M. Savelkoul, John W. Rossen, Jan Kluytmans

**Affiliations:** 1 Department of Infection Control, Amphia Hospital, Breda, the Netherlands; 2 Laboratory for Medical Microbiology and Immunology, Elisabeth-TweeSteden Hospital, Tilburg, the Netherlands; 3 Academy for Technology and Environmental Health, Avans University of Applied Sciences, Breda, the Netherlands; 4 University of Groningen, University Medical Center Groningen, Department of Medical Microbiology, Groningen, the Netherlands; 5 Maastricht University Medical Centre, Caphri School for Public Health and Primary Care, Department of Medical Microbiology, Maastricht, the Netherlands; 6 Amsterdam University Medical Center, Vrije Universiteit Amsterdam, Department of Medical Microbiology & Infection Control, Amsterdam, the Netherlands; 7 Julius Center for Health Sciences and Primary Care, University Medical Center Utrecht, Utrecht University, Utrecht, the Netherlands; Institut National de la Recherche Agronomique, FRANCE

## Abstract

Community-acquired carriage and infections due to extended-spectrum beta-lactamase producing *Enterobacteriaceae* (ESBL-E) are increasing worldwide, resulting in increased morbidity, mortality and healthcare costs. The origins of community-acquired ESBL-E carriage and infections remain unclear. Bean sprouts are a potential source of *Enterobacteriaceae* for the community, as illustrated by outbreaks of pathogenic *Enterobacteriaceae* in the past. The current study focuses on contamination of retail bean sprouts with ESBL-E in the Netherlands. Of 131 bean sprout samples purchased between 2013 and 2016, 25 (19%) were contaminated with ESBL-E. The detected isolates were almost exclusively *Klebsiella spp*. and co-resistance to other antibiotics was observed frequently. Over time there was substantial genetic diversity between isolates. On the other hand, isolates from samples closely matched in time were frequently clonally related, indicative of batch contamination. Remarkably, no *Escherichia coli* was found. In conclusion, bean sprouts frequently harbor ESBL-E, which is a potential source for consumers.

## Introduction

Over the past 15 years human carriage and infections due to antimicrobial resistant *Enterobacteriaceae* have increased substantially, and concomitantly the impact on morbidity, mortality and healthcare costs are rising [1–4]. Infections caused by extended-spectrum beta-lactamase producing *Enterobacteriaceae* (ESBL-E) originally were a hospital related problem, however, this has shifted to a largely community-acquired problem [5]. The reservoirs and transmission routes of community acquired ESBL-E are poorly understood and seem to be a multifactorial problem.

Risk factors for ESBL-E carriage can be classified as intrinsic and extrinsic. Intrinsic risk factors decrease the natural barriers of the body, such as decreased gastric acid production caused by proton-pump inhibitors or decreased colonization resistance due to antibiotic use [6–8]. Extrinsic risk factors largely entail the frequency and intensity of contact with ESBL-E. Travel to high endemic areas and contact with ESBL-E positive family members and pets are typical examples [9–12]. Many food items have been shown to contain ESBL-E and as such are potential sources for acquisition of ESBL-E by humans. In recent years meat has gained much interest as a potential source, but a large variety of food items are contaminated with ESBL-E, including vegetables and drinking water [13–20]. One study from the Netherlands and one from South-Korea reported ESBL-E on bean sprouts, among other vegetables [19,21]. This is relevant as bean sprouts are often consumed raw, and as such have a higher risk of transmission than food items that are cooked before consumption [22]. It has been shown in the past that bean sprouts carry the potential to be the source of large-scale community outbreaks with pathogenic *Enterobacteriaceae*, as was the case with *E*. *coli* O104:H4 causing hemolytic-uremic syndrome in Germany in 2011 [23,24]. The aim of the current study is to investigate to what extend bean sprouts in the Netherlands are contaminated with ESBL-E.

## Materials and methods

### Study design

Bean sprout samples were purchased from supermarkets and grocery stores (including: ethnic markets and green grocers) in the Netherlands from December 2013 until January 2016. For the ESBL-E prevalence survey a maximum of one bean sprout sample per store per day was included. For all the samples, the following variables were noted: store of purchase, date of purchase and if available the expiration date. Besides the samples for the prevalence survey, extra samples per store per day were obtained in the first sampling period (2013–2014) to determine the presence of batch contamination. Isolates from the additional samples were used only to determine the presence of batch contamination and were excluded from the other analyses.

### Microbiological methods

Per sample, twelve grams of bean sprouts were enriched in 15 mL tryptic soy broth (TSB). After overnight incubation, 100 μL of the TSB was transferred to a selective TSB, containing cefotaxime (0.25 mg/L) and vancomycin (8 mg/L) (TSB-VC). After overnight incubation, 10 μL of the TSB-VC was subcultured on an ESBL screening agar, EbSA (AlphaOmega, ‘s-Gravenhage, the Netherlands), consisting of a split McConkey agar plate containing cloxacillin (400 mg/L), vancomycin (64 mg/L) and either cefotaxime or ceftazidime (1 mg/L). Species identification (VITEK-MS, bioMérieux, Marcy l’Etoile, France) and antibiotic susceptibility testing (VITEK2, bioMérieux, Marcy l’Etoile, France) were performed for all oxidase-negative Gram-negative isolates that grew on the EbSA. Minimal inhibitory concentrations (MIC) are given in mg/L. The production of ESBL was phenotypically confirmed with the combination disk diffusion method for cefotaxime (30 μg), ceftazidime (30 μg) and cefepime (30 μg). All with and without clavulanic acid (10 μg) (Rosco, Taastrup, Denmark). Test results were considered positive if the diameter of the inhibition zone was ≥5 mm larger for the disk with clavulanic acid as compared to the disk without clavulanic acid [25,26]. For interpretation of the phenotypic susceptibility testing EUCAST clinical breakpoints–bacteria (v 7.1) was used [27].

### Whole genome sequencing (WGS), genome assembly and quality control (QC)

Phenotypically confirmed ESBL-E isolates were sequenced on a MiSeq (Illumina, San Diego, United States) and assembled with CLC Genomics Workbench 9.0, 9.0.1 or 9.5.2 (Qiagen, Hilden, Germany) as was previously described in more detail [28]. As quality control parameters, the following criteria were used: coverage: ≥ 30; number of scaffolds: ≤1000; N50: ≥ 15,000 bases and maximum scaffold length: ≥ 50,000 bases.

### Analyses of WGS data: species determination, resistance gene detection, Multi Locus Sequence Typing (MLST) and whole-genome MLST (wgMLST)

Assembled genomes were analyzed using an open access bioinformatics web tool (https://cge.cbs.dtu.dk/services/cge/, DTU, Copenhagen). This was done with ResFinder for analyses of resistance genes, PlasmidFinder for plasmid replicons and MLST 1.8 for MLST [29–31]. The services are combined in the bacterial analysis pipeline–batch upload mode [32]. This analysis pipeline also incorporates species determination with KmerFinder [33]. In case of conflicting results between the phenotypical MALDI-TOF and genetic KmerFinder 2.0, final species determination was based on the *rpoB* sequence [34].

wgMLST was performed using Ridom SeqSphere+, version 3.4.0 (Ridom, Münster, Germany). The species specific wgMLST typing schemes used in this study (*K*. *pneumoniae* and *K*. *oxytoca)* are described by Kluytmans–van den Bergh et al [28]. The pairwise genetic difference between isolates was calculated by dividing the number of allele differences by the total number of shared alleles from the typing scheme present in both sequences, using a pairwise ignoring missing values approach. Species-specific thresholds for relatedness were used [28]. Using pairwise comparisons, a distance matrix was built. The relatedness of the isolates was inferred using the Neighbor-Joining method [35]. The Neighbor-Joining trees were constructed using MEGA6 [36].

### Statistical analyses

Data were analyzed using Statistical Package for Social Science software (IBM SPSS Statistics 24.0, Armonk, NY). To test for differences in ESBL-E prevalence between supermarket chains and grocery stores and between the different supermarket chains, the Fischer exact test was used. As a measure of diversity between the isolates the Simpson Diversity Index (SID) was calculated based on MLST [37,38]. Confidence intervals of percentages were calculated with GraphPad QuickCalcs (GraphPad Software, La Jolla, California).

### Accession number

Raw sequencing reads were submitted to the European Nucleotide Archive of the European Bioinformatics Institute and are available under the study accession number PRJEB25080.

## Results

### ESBL-E prevalence in bean sprouts

A total of 131 bean sprout samples were tested for the presence of ESBL-E of which 25 (19.1%) tested positive (Table 1). The ESBL-E prevalence varied depending on the store of purchase. Between supermarket chains the largest difference in ESBL-E prevalence was between chains three and four, with a prevalence of 45.0% and 4.8%, respectively. In general, samples from supermarkets were more frequently contaminated with ESBL-E than samples from grocery stores (ESBL-E prevalence of 25.3% and 5.0% respectively, p = 0.007).

**Table 1:** ESBL-E prevalence in bean sprout samples in the Netherlands, 2013–2016.

	No. samples	ESBL-E positive (%)	95% CI	P
Prevalence survey samples	131	25 (19.1)	13.2–26.7	
Store of purchase (N = 131)				0.013
Supermarket chain 1	23	5 (21.7)	9.2–42.3	
Supermarket chain 2	23	7 (30.4)	15.4–51.1	
Supermarket chain 3	20	9 (45.0)	25.8–65.8	
Supermarket chain 4	21	1 (4.8)	<0.01–24.4	
Supermarket chain 5	4	1 (25.0)	3.4–71.1	
Grocery store 1	10	0 (0)	0.00–32.1	
Grocery store 2	10	1 (10)	<0.01–42.6	
Grocery store 3	10	1 (10)	<0.01–42.6	
Grocery store 4	10	0 (0)	0.00–32.1	
Supermarket (N = 131)				0.007
Yes	91	23 (25.3)	17.4–35.1	
No	40	2 (5)	0.5–17.4	

CI confidence interval, P p-value of the Fischer exact test.

*K*. *pneumoniae* was the predominant species (n = 21, 80.8%), followed by *K*. *oxytoca* (n = 3, 11.5%) and *K*. *variicola* (n = 1, 3.8%). One sample contained an ESBL-producing *E*. *cloacae* (3.8%) besides an ESBL-producing *K*. *pneumoniae*. ESBL-producing *E*. *coli* was not found.

Results of antimicrobial-susceptibility testing are shown in Tables 2 and 3. Besides the ESBL phenotype, high rates of resistance were found against ciprofloxacin (69.2%), trimethoprim-sulfamethoxazole (80.8%) and tobramycin (84.6%). Combined resistance against these three antibiotics was present in 50.0% of the isolates. Resistance against piperacillin-tazobactam was found in two of 26 isolates (7.7%). All isolates were susceptible to meropenem and colistin.

**Table 2:** Susceptibility profiles to different beta-lactams, detected extended-spectrum beta-lactamase (ESBL) genes, plasmid replicons and multi-locus sequence types (MLST) of ESBL-producing *Enterobacteriaceae* isolates from bean sprouts in the Netherlands.

Species ID	AMC	TZP	CTX	CAZ	ESBL genes	Plasmid replicons	MLST
***K*. *pneumoniae***							
13	8	≤4	4	≤1	blaCTX-M-14	IncFII,IncFIB(K),ColRNAI	ST-1296
5	16	8	8	2	blaCTX-M-3	IncFII(K)	ST-1565
19	4	≤4	≤1	≤1	blaSHV-2	IncFIA(HI1),IncFIB(K), IncHI1B,ColRNAI	ST-2176
16	8	≤4	8	≤1	blaCTX-M-14	IncFII,IncFIB(K),ColRNAI	ST-2657
6	16	32	≥64	4	blaSHV-2	IncFIA(HI1),IncR	ST-2658
20	16	16	8	2	blaCTX-M-3, blaSHV-99^a^	IncFIA(HI1),IncFIB(pKPHS1),IncFIB(K),IncFII(K),IncR,IncQ1,ColRNAI	ST-2659
21	16	8	8	2	blaCTX-M-3, blaSHV-99^a^	IncFIA(HI1),IncFIB(pKPHS1),IncFIB(K),IncFII(K),IncR,IncQ1,ColRNAI	ST-2659
18	16	8	32	≤1	blaSHV-2	IncFIA(HI1),IncR	ST-280
14	8	8	16	16	blaCTX-M-27	IncFIA(HI1),IncFIB(K)	ST-37
15	8	8	≥64	16	blaCTX-M-27	IncFIA(HI1),IncFIB(K)	ST-37
17	16	8	8	2	blaSHV-2	IncFIA(HI1),IncFII, IncFIB(K)	ST-39
8	8	≤4	≤1	≤1	blaSHV-2	IncFiA(HI1),IncR,ColRNAI	ST-392
7,9	4	≤4	≤1	≤1	blaSHV-2	IncFiA(HI1),IncR,ColRNAI	ST-392
11	16	≤4	8	≤1	blaSHV-2	IncFIA(HI1),IncR,ColRNAI	ST-45
12	8	≤4	≥64	8	blaCTX-M-15	IncFIB(K)	ST-45
10	8	8	2	2	blaSHV-2	IncFIA(HI1),Col(BS512),IncR, Col(MG828),ColRNAI	ST-485
4	16	8	≥64	≤1	blaCTX-M-3, blaSHV-27	IncFII(K),IncQ1	ST-661
1,2,3	16	≤4	8	≤1	blaCTX-M-3, blaSHV-27	IncFII(K),IncQ1	ST-661
***K*. *oxytoca***							
29	8	≤4	8	≤1	blaCTX-M-14	IncFIA(HI1), IncFIB(pKPHS1),IncN	ST-195
33	8	≤4	4	≤1	blaCTX-M-3	IncN,IncU	ST-196
32	8	≤4	≥64	4	blaCTX-M-27	IncFIA(HI1),IncR	ST-2
***K*. *variicola***							
34	8	≤4	8	≤1	blaCTX-M-3	IncN2,IncFIB(K),ColRNAI	ST-1142
***E*. *cloacae***							
31	≥32	8	≥64	4	blaCTX-M-15	IncFII(pECLA),IncFIB(pENTE01), IncFIB(pECLA),ColRNAI	ST-144

^a^ESBL genes called with a less than 100% identity and or length less than 100%. Minimum identity and length for call: 90.00% and 60% respectively. All plasmid replicons called by PlasmidFinder in default settings were reported. Shading within table indicates susceptibility interpretation according to EUCAST breakpoint table version 7.1, dark grey: resistant, light grey: intermediate and white: susceptible. ID isolate identification number, AMC amoxicillin-clavulanic acid, TZP piperacillin-tazobactam, CTX cefotaxim, CAZ ceftazidime, ESBL extended-spectrum beta-lactamase, MLST multilocus sequencing typing, ST sequence type.

**Table 3:** Detected genes associated with resistance to different classes of antibiotics and phenotypic susceptibility profiles to most of these classes of antibiotics.

Species ID	TOB	CIP	TMP	SXT	Aminoglycoside^†^	Quinolone	TMP	SUL	TET	MAC
***K*. *pneumoniae***										
13	8	0.5	≥16	≥320	aac(3)-IId^a^,strA,strB	oqxA^a^,oqxB^a^, QnrS1	dfrA1	sul1, sul2	tet(A), tet(D)	
5	8	≥4	≥16	≥320	aadA16^a^,aac(6')Ib-cr	oqxA^a^,oqxB^a^, QnrB49^a^,QnrS1	dfrA27	sul1	tet(A)^a^	mph(A)
19	8	1	≤0.5	≤20	aac(3)-IId^a^,strA,strB	oqxA^a^,oqxB^a^, QnrS1		sul2	tet(D)	
16	8	0.5	≥16	≤20	aac(3)-IId^a^	oqxA^a^,oqxB^a^, QnrS1	dfrA1	sul1	tet(A)	
6	8	≥4	≥16	≥320	aac(3)-IId^a^,aadA16^a^, strA^a^,strB^a^, aac(6')Ib-cr	oqxA^a^,oqxB^a^, QnrB6	dfrA27	sul1, sul2	tet(D)	
20,21	8	≥4	≥16	≥320	aac(3)-IId^a^,aadA16^a^, aph(3')-Ia^a^,strA,strB, aac(6')Ib-cr	oqxA^a^,oqxB^a^, QnrB49^a^,QnrS1	dfrA27	sul2	tet(A)^a^	mph(A)
18	8	0.5	8	40	aac(3)-IId^a^,aadA16^a^, strA^a^,strB^a^,aacA4^a^, aac(6')Ib-cr^a^	oqxA^a^,oqxB^a^, QnrB6	dfrA27	sul1, sul2	tet(D)	
14	2	≥4	≥16	≥320	aadA16^a^,aac(6')Ib-cr	oqxA,oqxB	dfrA27	sul1	tet(D)	
15	4	≥4	≥16	≥320	aadA16^a^,aac(6')Ib-cr	oqxA,oqxB	dfrA27	sul1	tet(D)	
17	8	0.5	≥16	≥320	aac(3)-IId^a^, strA^a^,strB^a^	oqxA^a^,oqxB^a^, QnrS1	dfrA14^a^	sul2	tet(A)^a^	mph(A)
7,8,9	8	≥4	≥16	≥320	aac(3)-IId^a^,aadA16^a^, aac(6')Ib-cr	oqxA^a^,oqxB^a^	dfrA27	sul1, sul2	tet(A)^a^, tet(D)	
11	8	≤0.25	≤0.5	≤20	aac(3)-IId^a^, strA^a^,strB^a^	oqxA^a^,oqxB^a^, QnrB6		sul1, sul2	tet(D)	
12	≤1	1	≥16	≥320	strA,strB	oqxA^a^,oqxB^a^, QnrS1	dfrA14^a^	sul2		
10	8	≥4	≥16	≥320	aac(3)-IId^a^,aadA16^a^, aac(6')Ib-cr	oqxA^a^,oqxB^a^, QnrB49^a^	dfrA27	sul1, sul2	tet(A)^a^, tet(D)	
4	8	2	≥16	≥320	aac(3)-IId^a^,aadA16^a^, aph(3')-Ia^a^,strA,strB, aac(6')Ib-cr	oqxA^a^,oqxB^a^, QnrB49^a^,QnrS1	dfrA27	sul1, sul2	tet(A)^a^	mph(A)
1,2,3	8	2	≥16	≥320	aac(3)-IId^a^,aadA16^a^, aph(3')-Ia^a^,strA,strB, aac(6')Ib-cr	oqxA^a^,oqxB^a^, QnrB49^a^,QnrS1	dfrA27	sul1, sul2	tet(A)^a^	mph(A)
***K*. *oxytoca***										
29	≤1	≤0.25	≤0.5	≤20	aph(3')-Ia^a^					
33	8	0.5	≥16	≥320	strA^a^,strB^a^,aacA4, aac(6')Ib-cr^a^	QnrS1	dfrA14^a^	sul2		
32	8	≥4	≥16	≥320	aac(3)-IId^a^,aadA16^a^, aac(6')Ib-cr	QnrB52	dfrA27	sul1	tet(A)^a^	
***K*. *variicola***										
34	≤1	≤0.25	≤0.5	≤20		oqxA^a^,oqxB^a^				
***E*. *cloacae***										
31	8	0.5	≥16	≥320	aac(3)-IIa^a^, aadA1^a^,strA,strB, aac(6')Ib-cr	QnrB1^a^	dfrA14^a^	sul2	tet(A)^a^	

^a^Genes called with a less than 100% identity and or length less than 100%. Minimum identity and length for call: 90.00% and 60% respectively.

^**†**^aac(6')Ib-cr confers resistance to aminoglycosides and quinolones.

Shading within table indicates susceptibility interpretation according to EUCAST breakpoint table version 7.1, dark grey: resistant, light grey: intermediate and white: susceptible. ID isolate identification number, TOB tobramycin, CIP ciproflacin, TMP trimethoprim, SXT trimethoprim-sulfamethoxazole, SUL sulphonamide, TET tetracyclin, MAC macrolide.

### Genetic characteristics of ESBL-E isolated from bean sprouts

Quality control results and recoded file names to access files from ENA are displayed in S1 Table and S2 Table respectively. All of the phenotypically confirmed ESBL-E isolates contained at least one ESBL gene (Table 2). The following ESBL genes were detected: in 9 (34.6%) isolates the *bla*_SHV-2_ gene, in 5 (19.2%) the *bla*_CTX-M-3_ gene, in 4 (15.4%) both the *bla*_SHV-27_ gene and *bla*_CTX-M-3_ gene, in 3 (11.5%) the *bla*_CTX-M-14_ gene, in 3 (11.5%) the *bla*_CTX-M-27_ gene and in 2 (7.7%) the *bla*_CTX-M-15_ gene. In two isolates containing the *bla*_CTX-M-3_ gene, the *bla*_SHV-99_ gene was also detected, with one mismatching nucleotide. The most frequently detected plasmid replicons as reported by PlasmidFinder 1.2 were IncFIA, IncFIB, IncFII, Col and IncR (Table 2). Most of the plasmid replicons called by PlasmidFinder were variants on the genes in the database [31]. MLST results of the isolates are shown in Table 2. Unknown MLST types were submitted to the corresponding databases. Three new MLST types were added for *K*. *pneumoniae* (ST2657, ST2658 and ST2659) and two were added for *K*. *oxytoca* (ST195 and ST196). The Simpson index of diversity (1-D) based on MLST was 0.96, 95% CI 0.92–1.00, demonstrating a high diversity between the isolates. Genes associated with resistance to aminoglycosides, quinolones, trimethoprim, sulphonamides, tetracyclines and macrolides were frequently present (Table 3).

### wgMLST

**Fig 1:**
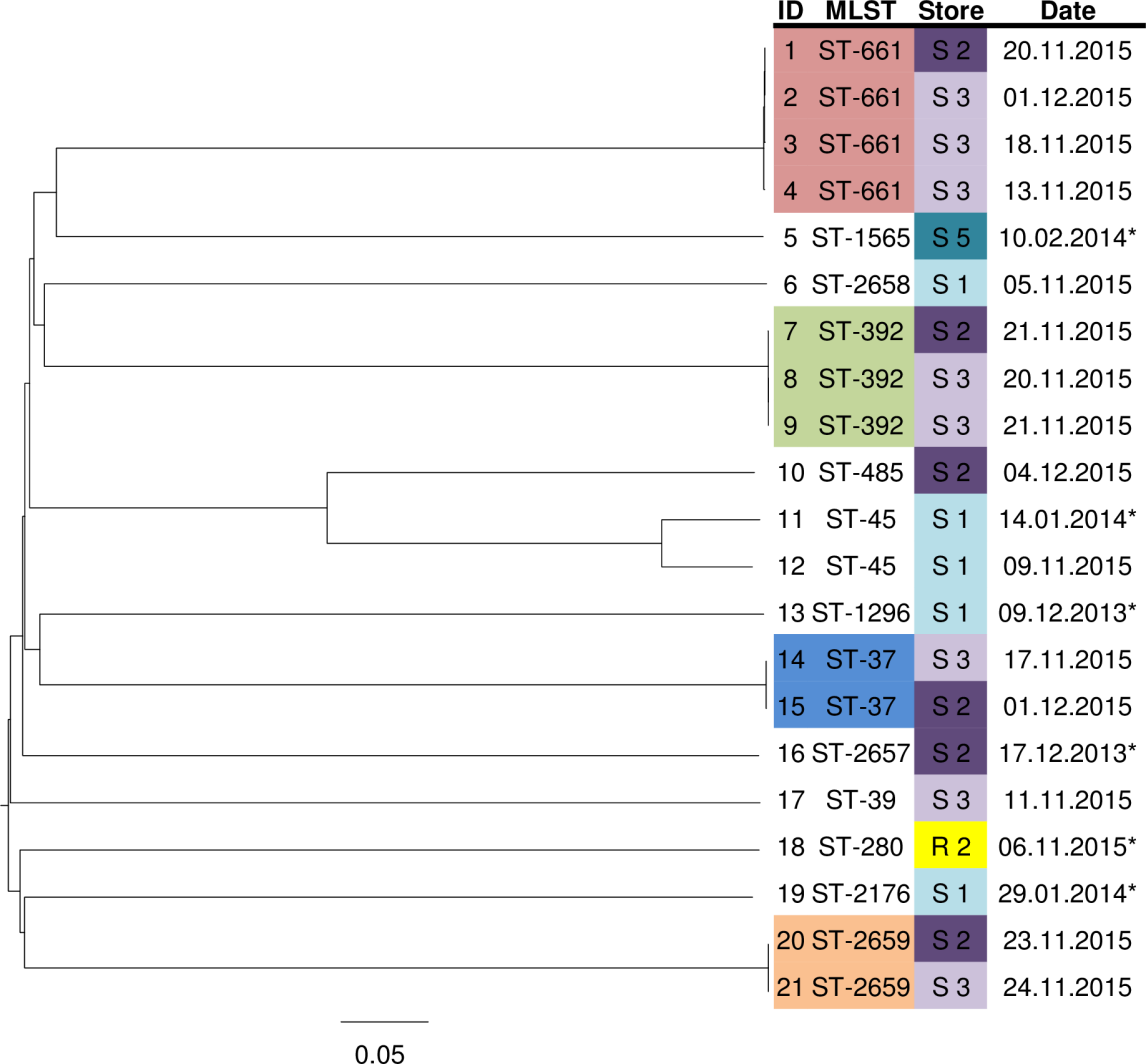
Neighbor-joining tree based on the wgMLST analysis of *K*. *pneumonia* isolates from the prevalence survey of bean sprout samples (2013–2016), using a pairwise ignore missing values approach. Scale shows genetic distance. wgMLST clusters are color coded (ID and MLST) as is the store of purchase. The date is the expiration date unless labeled with * in which case date of purchase of the sample is depicted. ID isolate identification number, MLST multilocus sequence typing, ST sequence type, S supermarket chain, R retailer.

The genetic relatedness of the 21 *K*. *pneumoniae* isolates from the prevalence survey is shown in Fig 1. Isolates were either clonally related or had large genetic diversity. The median genetic distance of *K*. *pneumoniae* isolate-to-isolate comparisons was 0.0002; range, 0.0000–0.0016 for clonally related isolates (n = 11), and 0.8524; range 0.1082–0.8721 for non-clonally related isolates (n = 199). Four clusters were identified; one cluster consisted of four isolates, one cluster contained three isolates and two clusters contained two isolates. Isolates within clusters came from samples that had expiration dates closely matched in time. The longest time between expiration dates within a cluster was 18 days. Clustering isolates were detected in samples purchased from different supermarkets. wgMLST analysis of the three *K*. oxytoca isolates from the prevalence study revealed no clonal relatedness. The smallest genetic distance was between isolates 29 and 33, which was 0.71. The genetic distance between isolates 29 and 32 and between 32 and 33 were both 0.99.

### Batch contamination of bean sprout samples

**Fig 2:**
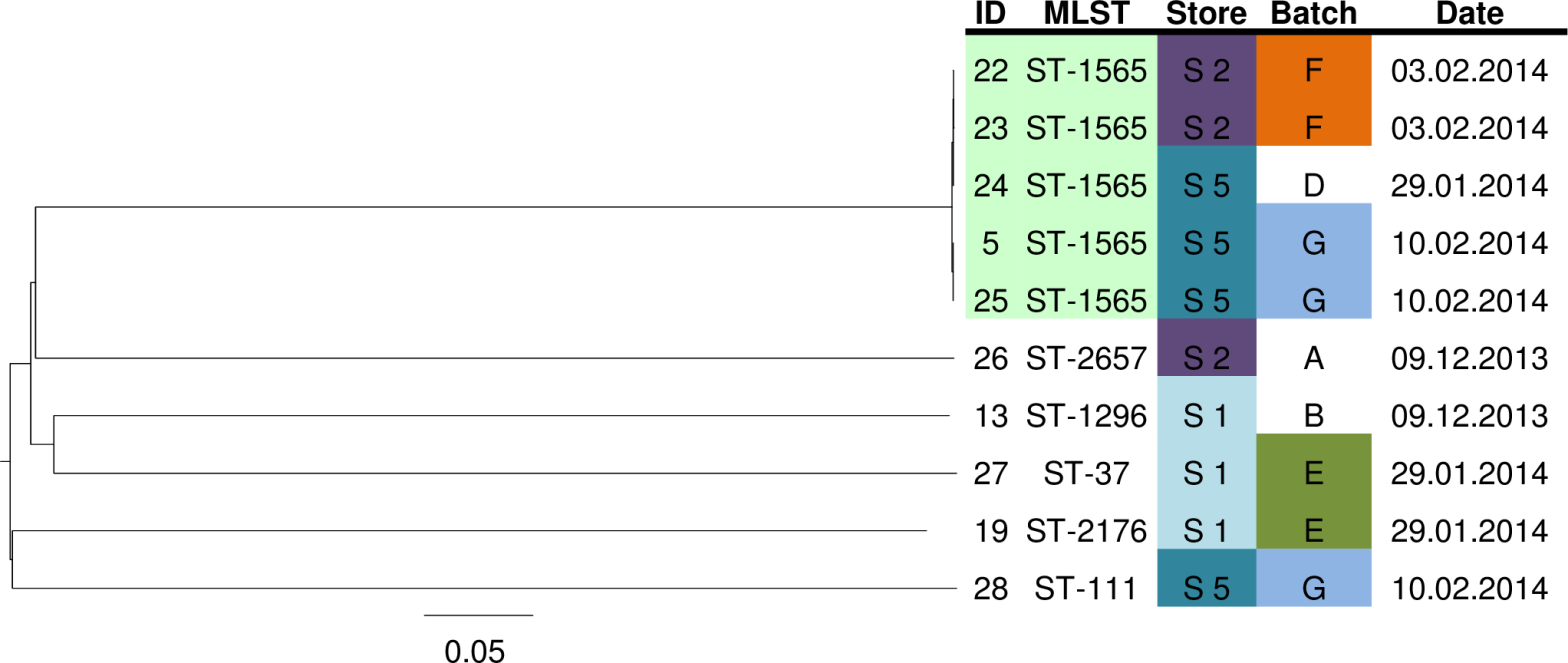
Neighbor-joining tree based on the wgMLST analysis of *K*. *pneumonia* isolates from multiple batches of bean sprouts, using a pairwise ignore missing values approach. Scale shows genetic distance. A batch was defined as bean sprout samples purchased on the same day from the same supermarket chain. wgMLST clusters are color coded (ID and MLST) as are store and batch of purchase. The date is the date of purchase of the sample. ID isolate identification number, MLST multilocus sequence typing, ST sequence type, S supermarket chain, R retailer.

In the period from December 2013 until March 2014 additional samples were purchased from different supermarkets, to investigate the occurrence of batch contamination with ESBL-E. Twenty-seven samples, coming from seven batches; three batches consisted of two samples, three batches consisted of five samples and one batch consisted of six samples. Analyzing the samples in a batch-by-batch manner shows three separate batch contamination events: two batches with *K*. pneumoniae (batch F and G) and one with *K*. *oxytoca* (batch F; Table 4). When comparing the 10 *K*. *pneumoniae* isolates from the batch contamination experiment with each other using wgMLST, contamination of five samples with one clone was found (Fig 2). This cluster occurred over a time period of thirteen days and was spread over two supermarket chains.

**Table 4:** Presence of ESBL-E in bean sprout samples purchased from supermarkets in batches.

Batch	No. samples in batch	No. samples ESBL-E positive (%) in batch	Clones present in >1 sample in batch	No. samples with clone and species	MLST of clone	Date of purchase	Store	ID
A	2	1 (50)	no	*1x K*. *pneumoniae*	ST-2657	09/12/13	2	26
B	2	1 (50)	no	*1x K*. *pneumoniae*	ST-1296	09/12/13	1	13
C	2	0 (0)	no	*-*		17/12/13	5	
D	5	1 (20)	no	*1x K*. *pneumoniae*	ST-1565^a^	29/01/14	5	24
E	5	2 (40)	no	*1x K*. *pneumoniae*	ST-2176	29/01/14	1	19
			no	*1x K*. *pneumoniae*	ST-37			27
F	5	4 (80)	yes	*2x K*. *oxytoca*	ST-195^a^	03/02/14	2	29,30
			yes	*2x K*. *pneumoniae*	ST-1565^a^			22,23
G	6	3 (50)	yes	*2x K*. *pneumoniae*	ST-1565^a^	10/02/14	5	5,25
			no	*1x K*. *pneumoniae*	ST-111			28

^a^ Isolates with the same ST are also clonally related on basis of wgMLST analyses. ESBL-E, extended-spectrum beta-lactamase producing *Enterobacteriaceae*, MLST multilocus sequence typing, ST sequence type, isolate identification number.

## Discussion

To our knowledge this is the first study focusing specifically on the ESBL-E prevalence in retail bean sprouts to date. An ESBL-E prevalence of 19% was found, being almost exclusively ESBL-producing *Klebsiella* spp. No ESBL-producing *E*. *coli* were found. The ESBL-E isolates found over time were either genetically highly diverse or clearly within the thresholds of clonal relatedness between epidemiologically related isolates as described by Kluytmans–van den Bergh et al [28]. The clonally related isolates always came from samples that were purchased within weeks of each other, which is suggestive for batch contamination. These findings indicate that there is a continuous influx of unrelated ESBL-E isolates and no prolonged persistence of specific clones.

A remarkable finding is that 96.2% of the isolates are of the genus *Klebsiella*, 80.8% being *K*. *pneumoniae*. The complete absence of ESBL-producing *E*. *coli* is noteworthy. We are unaware of factors favoring the growth of *K*. *pneumoniae* or suppressing the growth of other pathogens in the bean sprout production process. Other studies that present data on ESBL-E from bean sprouts reported similar high percentages of *K*. *pneumoniae*, namely, 80% and 84% in the studies by Reuland et al. (the Netherlands) and Kim et al. (South Korea) respectively [19,21].

Besides the resistance to beta-lactams, we found high levels of co-resistance to important antimicrobial agents like ciprofloxacin, trimethoprim-sulfamethoxazole and tobramycin. Combined resistance to these three antibiotics and the ESBL phenotype was present in 50% of the isolates. This rate of co-resistance is higher than what is found in ESBL-E isolates from human carriage, namely 12% [39]. Combined resistance to the three antibiotics and the ESBL phenotype for *K*. *pneumoniae* from blood cultures in four peripheral hospitals in the South of the Netherlands was 18.3% (11 of 60 isolates, time period 1-1-2010–1-1-2018, unpublished data). None of the isolates from the current study showed resistance to carbapenems or colistin.

The ESBL-genes detected in bean sprouts have also been detected in the human population. For instance the *bla*_CTX-M-15_ and *bla*_CTX-M-14_ genes, which were detected in bean sprouts in the current study, are among the most frequently detected ESBL genes in human carriage and bloodstream infections in the Netherlands [40–42]. Also *bla*_CTX-M-3_ and *bla*_CTX-M-27_ have been reported to be present in bloodstream infections in the Netherlands [42]. The most commonly detected ESBL gene in the current study, *bla*_*SHV-2*_, has also been reported to be present in clinical samples [40]. However, the *bla*_*SHV*_ genes are not always typed to the specific variants within the group, making comparison of the exact SHV types difficult [41,43]. Although similar ESBL genes are found in bean sprouts and humans, it is difficult to judge the impact of the ESBL-gene reservoir in bean sprouts on humans, as this study was not designed to make a direct comparison. However, bean sprouts have the potential to spread *Enterobacteriaceae* to humans, as has been shown by multiple outbreaks of pathogenic *Enterobacteriaceae* in humans in the past [23,24,44]. Therefore, our findings should be considered as a potential threat for humans but based on the current information we cannot quantify the size of the effect.

Our study has some strengths and limitations. Strengths of the study are the fact that samples were taken in different time periods, showing that ESBL-E contamination of bean sprouts is not an incidental finding. In addition, sensitive culture techniques were employed, using broth enrichment and a validated ESBL screening agar [45]. Furthermore, genetic confirmation of ESBL genes and precise typing and clustering methods using whole genome sequencing were used.

A limitation of the study is that we did not use a (semi-) quantitative culture method to quantify the load of the ESBL-E in bean sprouts. This information could have been important to estimate the possible impact for humans [22].

A further limitation of the study is the lack of information on the label of the bean sprouts. For instance, no information on place of production or production batches was given on the label and expiration dates were not always present. For the analyses, batches were defined as samples purchased from the same supermarket chain or store, with the same expiration date or date of purchase, depending on the availability of the information. No tracking codes or batch numbers from producers were present on the packages, which would have allowed for more precise analyses. Care was taken to achieve a representative sample of bean sprouts sold in the Netherlands and minimize the effect of batch contamination on the reported ESBL-E prevalence.

A final limitation of the study was the analysis of the plasmid content of the detected isolates. PlasmidFinder results are reported which enable comparisons to other studies, but further analyses into the epidemiology of the plasmids would have been a valuable addition. However, unraveling plasmid DNA from chromosomal DNA from the available short-read sequencing data is still a conundrum and beyond the scope of this study.

Despite these caveats several conclusions can be made on the ESBL-E prevalence of the different stores. First, there are differences in ESBL-E prevalence between supermarket chains. Supermarket chain one, two and three have an ESBL-E prevalence of more than 20%, whereas supermarket chain four has a prevalence of 4.8%. The bean sprouts sold by supermarket chain two and three are likely to have an overlapping origin, as clonality in isolates from these supermarkets is common. Supermarket chain five was discontinued during the study period explaining the small number of samples. Second, a higher ESBL-E prevalence was found in samples from supermarkets compared to those from smaller retailers. The underlying causes of the differences in ESBL-E prevalence in the different stores are unknown. This may be caused by differences in the production process, network of transportation and differences in production scales with intrinsic possibilities of cross-contamination. Further studies are warranted to reveal the causes of contamination to decrease the overall ESBL-E prevalence in bean sprouts.

A final point on the ESBL-E contamination of bean sprout samples is the presence of batch contamination. High genetic variability of the ESBL-E isolates was seen in the bean sprout samples over time. In contrast, from samples purchased within three weeks of each other clonally related isolates were frequently cultured. Furthermore, in the experiment focusing on batch contamination, a single wgMLST clone was found in five different packages purchased from two different supermarket chains (Figs 1 and 2). These two observations support the hypothesis of batch contamination, however, not all samples from these batches were ESBL-E positive. This may indicate a varying load of ESBL-E or overgrowth with abundantly present non-fermenting bacteria. Also, the previously mentioned limitation of the definition of a batch may play a role.

In conclusion, 19.1% of bean sprout samples are contaminated with ESBL-E, with a remarkable high percentage of *Klebsiella* isolates in the absence of *E*. *coli*. The isolates are resistant to several other classes of antibiotics and are genetically highly diverse over time. Therefore, bean sprouts are a possible community source of ESBL-producing *Klebsiella spp*. Further investigations to the points of entry of ESBL-E into the production process and countermeasures against these entry points are warranted. Unfortunately, research looking into food as a vehicle for the spread of antimicrobial resistance in humans is greatly hampered by the lack of transparency in the food production process. In the current article the factory or even country of origin of the samples was not traceable for the researchers. We strongly suggest working towards a situation where basic information such as which country or countries the products were produced are clearly marked on the food items.

## Supporting information

S1 Table Summary of the quality control parameters of the WGS assemblies used in the study. (DOCX)

S2 Table Recoding of isolate names for correspondence of fastq files on the ENA site. (DOCX)
