# Supplementary material for: Extended-spectrum beta-lactamase producing Enterobacteriaceae (ESBL-E) isolated from bean sprouts in the Netherlands
Source: PLoS One. 2018 Aug 30;13(8):e0203338. doi: 10.1371/journal.pone.0203338 (PMC6117087; doi:10.1371/journal.pone.0203338)
Supplement: S1 Table — (DOCX) [file pone.0203338.s001.docx]

**S1 Table. Summary of the quality control parameters of the WGS assemblies used in the study.**

|  | **Mean** | **Median** | **Minimum** | **Maximum** |
| --- | --- | --- | --- | --- |
| **K. pneumoniae (n = 28)** |  |  |  |  |
| No. Scaffolds, *n* | 58.4 | 53.0 | 35 | 99 |
| N50, *n* | 291716.1 | 295293.5 | 142946 | 426749 |
| Max. scaffold length, *n* | 659241.7 | 685594.5 | 416351 | 915597 |
| Genome size, *n* | 5389681.1 | 5364165.0 | 5222704 | 5572072 |
| Coverage, *n* | 69.2 | 72.3 | 31.5 | 98.2 |
| Percentage of reads used, % | 97.5 | 97.8 | 94.5 | 98.7 |
| **K. oxytoca (n = 4)** |  |  |  |  |
| No. Scaffolds, *n* | 78.8 | 74.0 | 73 | 94 |
| N50, *n* | 181404.3 | 181331.0 | 138416 | 224539 |
| Max. scaffold length, *n* | 504542.5 | 493858.5 | 417814 | 612639 |
| Genome size, *n* | 6054428.0 | 6059100.5 | 5871338 | 6228173 |
| Coverage, *n* | 61.2 | 61.7 | 55.1 | 66.2 |
| Percentage of reads used, % | 97.9 | 98.2 | 96.9 | 98.5 |
| **K. variicola (n = 1)** |  |  |  |  |
| No. Scaffolds, *n* | 77.0 | 77.0 | 77 | 77 |
| N50, *n* | 162401.0 | 162401.0 | 162401 | 162401 |
| Max. scaffold length, *n* | 423756.0 | 423756.0 | 423756 | 423756 |
| Genome size, *n* | 5673740.0 | 5673740.0 | 5673740 | 5673740 |
| Coverage, *n* | 61.6 | 61.6 | 61.6 | 61.6 |
| Percentage of reads used, % | 97.5 | 97.5 | 97.5 | 97.5 |
| **E. cloacae (n = 1)** |  |  |  |  |
| No. Scaffolds, *n* | 53.0 | 53.0 | 53 | 53 |
| N50, *n* | 298624.0 | 298624.0 | 298624 | 298624 |
| Max. scaffold length, *n* | 625903.0 | 625903.0 | 625903 | 625903 |
| Genome size, *n* | 4710742.0 | 4710742.0 | 4710742 | 4710742 |
| Coverage, *n* | 105.0 | 105.0 | 105.0 | 105.0 |
| Percentage of reads used, % | 98.3 | 98.3 | 98.3 | 98.3 |
| N50 is the shortest scaffold length such that 50% of the entire assembly is contained in scaffolds equal to or larger than this length, max. maximum | | | | |
