# Supplementary material for: Extended-spectrum beta-lactamase producing Enterobacteriaceae (ESBL-E) isolated from bean sprouts in the Netherlands
Source: PLoS One. 2018 Aug 30;13(8):e0203338. doi: 10.1371/journal.pone.0203338 (PMC6117087; doi:10.1371/journal.pone.0203338)
Supplement: S2 Table — (DOCX) [file pone.0203338.s002.docx]

**S2 Table. Recoding of isolate names for correspondence of fastq files on the ENA site.**

| **Sample ID** | **Names of fastq files** | **Present in prevalence study** | **Batch number** |
| --- | --- | --- | --- |
| 1 | 15M105753-1 | yes |  |
| 2 | 15M109131-1 | yes |  |
| 3 | 15M104880-1 | yes |  |
| 4 | 15M102484-1 | yes |  |
| 5 | 14M013120-1 | yes | G |
| 6 | 15M100995-2 | yes |  |
| 7 | 15M105754-1 | yes |  |
| 8 | 15M104882-1 | yes |  |
| 9 | 15M105761-1 | yes |  |
| 10 | 15M110134-1 | yes |  |
| 11 | 14M004312-1 | yes |  |
| 12 | 15M101835-3 | yes |  |
| 13 | 13M104391-1 | yes | B |
| 14 | 15M103835-2 | yes |  |
| 15 | 15M109129-1 | yes |  |
| 16 | 13M106860-4 | yes |  |
| 17 | 15M101900-2 | yes |  |
| 18 | 15M101907-2 | yes |  |
| 19 | 14M009417-2 | yes | E |
| 20 | 15M105756-1 | yes |  |
| 21 | 15M105764-1 | yes |  |
| 29 | 14M010914-7 | yes | F |
| 31 | 15M101907-1 | yes |  |
| 32 | 15M103834-1 | yes |  |
| 33 | 15M112937-1 | yes |  |
| 34 | 16M002405-1 | yes |  |
| 22 | 14M010915-5 |  | F |
| 23 | 14M010919-3 |  | F |
| 24 | 14M009431-3 |  | D |
| 25 | 14M013121-1 |  | G |
| 26 | 13M104441-1 |  | A |
| 27 | 14M009420-2 |  | E |
| 28 | 14M013125-1 |  | G |
| 30 | 14M010921-6 |  | F |
| Sample ID sample identification number, | | | |
